# Supplementary material for: Objective sleep assessment in >80,000 UK mid-life adults: Associations with sociodemographic characteristics, physical activity and caffeine
Source: PLoS One. 2019 Dec 27;14(12):e0226220. doi: 10.1371/journal.pone.0226220 (PMC6934314; doi:10.1371/journal.pone.0226220)
Supplement: Attachment — Submitted filename: Editor comments and responses.docx [file pone.0226220.s001.docx]

**Objective sleep assessment in >80,000 UK mid-life adults: associations with sociodemographic characteristics, physical activity and caffeine**

Dear Yinglin Xia,

We would like thank the reviewer for their time and helpful comments. Amendments to the original text can be identified using track.

Please find our responses to the comments as follows.

Editor comments:

1. No statistical model was previously carried out. Additional explanation on the statistical tests used are added in the method section.
2. More explanation on the z-test are added – z-test was used instead of standard t-test to determine whether 2 population mean was different because of the large sample size in the current study.
3. Details of the statistically analysis are now added as requested – Monte carlo method with 99% confidence level was used to estimate the exact significant level. ANOVA was used on continuous variables and Chi-square test was used on categorical variables. Kolmogorov-smirnov test indicates that sleep duration does not follow a normal distribution (D(84411)=0.065, p<0.001).
4. Statistical model results are added as suggested.
5. 11.6% (n=10918) were excluded from the current study including 1.6% (n=1425) due to participant drop-outs over-time. Comparison between the population in the current study and those excluded was undertaken, there are no significant differences in terms of age and gender, and therefore we believe that there is unlikely to be a selection bias.

Reviewer 1 comments:

**Letters denoting column proportion z-test in note of Table 1 are confusing, better if authors can explain them more.**

Additional explanation is added in the method section as suggested.

**Line 174-320: duplicate reference**

This might be an error in printing from Microsoft word. When viewing on the computer or print from PDF, this problem does not occur.

**Line 166-174: font and size need to edit**

This might be an error in printing from Microsoft word. When viewing on the computer or print from PDF, this problem does not occur.

**Line 84: “the oldest old”?**

This refers to the 85+ population.

**Line 56: “Difficulties” in?**

This refers to difficulties in initiating and maintaining sleep. The sentence has been amended to make it clearer.

**Line 93: age “40-69”; line 31: aged “43-69”?**

There is a time lag between self-reported data and accelerometry data. Participants were aged 40-69 years during in baseline assessment around 2007-2010, but their age are between 43-79 years when accelerometry data were taken around 2013-2015.

**Line 166-168: compared to what? Refer to Fig 1 or Table?**

This sentence has now been rearranged to improve clarity.

**Line 330: gender p-value: p<0.001 in table 2**

This typo has now been changed.

Reviewer 2 comments:

**Page 9-16, lines 166-320, large font texts and references list are incorrectly inserted here. Please check and correct them.**

This might be an error in printing from Microsoft word. When viewing on the computer or print from PDF, this problem does not occur.

**Are there any correlations between sleep time, measured by objective measures, and subjective sleepiness or sleep complaints?**

A previous study by Chaput JP et al. (2013) found a ‘U-shaped’ association between subjective sleep duration and metabolic measures and the current study found a similar association between that and objective sleep duration.

**Can actigraphy differentiate sleep and immobile waking state?**

Vincent T. van Hees et al. (2015) found that GGIR over-estimates sleep duration by 31 minutes with 83% accuracy. If arm angle does not change greater than 5 degrees in 5 minutes then it is considered a bout of sleep. Therefore actigraphy remains an acceptable tool to estimate the major sleep period, widely used across a number of different population cohort studies.

**Information about smoking and alcohol intake can interfere with sleep and should be presented if possible.**

Smoking status and alcohol intake information are available, but due to the ~5 years’ time lag between these data and accelerometry data, smoking and alcohol data is not presented in this paper.

**Statistical analysis should be described in more details, as this study address a large sample.**

More information on the statistical tests are added in the method section as suggested.

Yours sincerely

Miss Gewei Zhu and Dr Kirstie Anderson
